# Effect of traditional Chinese exercises on the physical and mental health of stroke patients: a meta-analysis

**DOI:** 10.3389/fneur.2024.1455679

**Published:** 2024-10-16

**Authors:** Yan Shao, Jing-Yuan Han, Hai-Long Li, Zhu-Ping Ren, Hui Yang

**Affiliations:** ^1^China Academy of Athletics, Beijing Sport University, Beijing, China; ^2^Chinese Exercise for Life Enhancement Division, Chinese Martial Arts College, Beijing Sport University, Beijing, China

**Keywords:** Baduanjin, Qigong, sports rehabilitation, stroke, Tai Chi

## Abstract

**Background:**

This meta-analysis aimed to investigate the effects of traditional Chinese exercises on stroke and explore the dose-response relationship between the type of exercise and exercise duration with respect to physical and mental health.

**Methods:**

We searched PubMed, Web of Science, EBSCO, China National Knowledge Infrastructure (CNKI), Wan Fang Database, and China Science and Technology Journal Database to identify published randomized controlled trials (RCTs) related to stroke patients and traditional Chinese exercise that met the evaluation criteria, from the inception of the database until September 2022. After assessing the quality of the literature, we used RevMan5.4 for data analysis.

**Results:**

A total of 27 RCTs were included. The results of the meta-analysis revealed that motor function [MD = 4.79, 95% CI: (4.14, 5.43), *p* < 0.001], balance function [MD = 5.25, 95% CI: (3.93, 6.56), *p* < 0.001], and mental health [MD = −2.42, 95% CI: (−3.92 to −0.92), *p* = 0.002] were significantly better in the experimental group than in the control group.

**Conclusion:**

Traditional Chinese exercises have a positive effect on the physical and mental health of stroke patients, with the most significant benefit observed in balance function. While traditional Chinese exercises improve exercise capacity and mental health in stroke patients, these improvements are not directly correlated with longer practice time, and there appear to be certain limitations regarding duration.

## 1 Introduction

Stroke is one of the leading causes of mortality and disability worldwide (1) and, along with ischemic heart disease (IHD), accounted for nearly 1 in 4 deaths in 2010 (1). Although mortality rates due to stroke have generally declined in recent decades, owing to both a decrease in stroke incidence and lower fatality rates, 6.7 million people globally still died from stroke in 2012 (2). The growing proportion of stroke survivors between the ages of 20 and 64 further highlights the importance of preventive measures (3).

Multiple complex risk factors contribute to the incidence of stroke. While some factors, such as age, gender, ethnicity, or heredity, are non-modifiable, most risk factors are lifestyle-related and largely modifiable (4).

A study indicated that 10 potentially modifiable risk factors are collectively associated with 90% of the population attributable risk (4), with the major risk factor being hypertension, followed by factors such as hyperlipidemia, diabetes, unhealthy diet, overweight and/or obesity, tobacco use, excessive alcohol consumption, drug abuse, and physical inactivity (5).

According to a Centers for Disease Control and Prevention (CDC) study, one in two US adults was found to have hypertension based on blood pressure measurements; however, one-third of those individuals had not received an appropriate diagnosis or treatment (6), highlighting the need for improved treatment measures and evaluation of blood pressure lowering interventions.

Controlling modifiable risk factors is key to reducing the risk of stroke, with exercise being one of the most frequently recommended interventions due to its association with reductions in body weight (7), blood pressure (6), triglycerides (8), and improved glucose regulation (9). While conventional exercise interventions usually include aerobic, strength, or flexibility training, alternative exercise practices such as Tai Chi and Qigong have recently gained popularity for disease prevention (10).

Traditional Chinese exercises are mind-body practices that originated in ancient China over a thousand years ago. These practices involve coordinated gentle movements, relaxation through meditation, and controlled breathing techniques, including forms such as Tai Chi, Qigong, Baduanjin, Wuqinxi, Yijinjing, and Liuzijue (11).

Furthermore, these practices have been shown to lower blood pressure, manage complications, and reduce mortality (12). Traditional Chinese exercises are believed to promote balance by facilitating the flow of vital energy, or Qi, within the body, helping to alleviate energy blockages (13). This process contributes to both physical and psychological wellbeing. Additionally, these exercises fall within the domain of sports medicine, where an experienced practitioner transmits Qi to a patient to help treat various illnesses (14).

As per Lauche et al. (15) “No recommendation for or against the use of Tai Chi or Qigong for the primary prevention of stroke can be given at the current time. However, Tai Chi and Qigong show some potential in reducing some major stroke risk factors, and as such, more high-quality studies are required for a conclusive judgment on the efficacy and safety of Tai Chi and Qigong for healthy and risky populations”.

A systematic review and meta-analysis conducted by Wang et al. (16) suggest that Qigong could be a promising intervention for improving global cognitive function, executive function, memory, visuospatial ability, and cognitive processing speed in patients with neurological disorders. However, a larger sample size and stronger high-quality trials are required to draw more reliable conclusions regarding the efficacy of Qigong on cognition in people with other neurological disorders to recommend it as an intervention.

Yuen et al. (17) found that Qigong training was effective in improving balance, leg muscle strength, and mobility among people with chronic stroke compared to traditional fitness training. Qigong can be considered a safe and sustainable form of exercise that can be integrated into stroke rehabilitation programs. Zheng et al. (18) showed that Qigong exercise led to improved trunk control ability, respiratory muscle functions, and daily living activities among patients in the early recovery stage from stroke, compared to conventional respiratory training.

Based on the above conclusions, it can be concluded that Qigong is not always effective in the treatment of stroke. Therefore, in this study, we aim to search for randomized controlled trials (RCTs) on traditional Chinese exercises for stroke across six databases and conduct a meta-analysis to determine the magnitude and quality of the influence that traditional Chinese exercises have on stroke treatment.

## 2 Methods

### 2.1 Aims

This meta-analysis aimed to investigate the effects of traditional Chinese exercises on stroke and explore the dose-response relationship between exercise type and duration with respect to physical and mental health.

### 2.2 Study search

RCTs examining the effects of traditional Chinese exercises on stroke recovery were searched in the Web of Science Core Collections, EBSCO, China National Knowledge Infrastructure (CNKI), VIP, and Wanfang databases from their inception until 17 September 2022.

The search strategy combined subject-specific and free-text keywords, determined after repeated pre-checks, supplemented by manual searches, and tracing references of included literature where necessary. The English search terms and formulas used were as follows:

TS = ((after stroke OR post stroke OR after apoplexy OR after cerebral infarction OR ischemic stroke) AND (wushu OR martial arts OR Kung Fu OR baduanjin OR wuqinxi OR yijinjing OR liuzijue OR daoyin OR qigong OR tai chi OR sport OR exercise)).

### 2.3 Inclusion criteria

#### 2.3.1 Study participants

Included individuals who participated in RCTs assessing the impact of traditional Chinese exercises on the physical and mental health of stroke patients.

#### 2.3.2 Experimental participants

Patients who met the diagnostic criteria for stroke, as determined by both traditional Chinese and Western medicine, and who were diagnosed with cerebral infarction or cerebral hemorrhage using cranial CT or MRI. These patients were required to be in stable condition with no acute exacerbation within the 6 months before trial enrollment.

#### 2.3.3 Intervention method

The experimental group participated in traditional Chinese exercises (including Tai Chi, Qigong, and so on) in addition to routine rehabilitation. The control group was provided with only routine rehabilitation, which included routine nursing care, conventional physiotherapy, and conventional exercise therapy.

### 2.4 Exclusion criteria

Repeatedly published studies.

Literature that could not be extracted from raw data.

Literature that could not be retrieved in full text.

Literature with poor methodological quality and imprecise experimental design (graded C).

Studies where the type of study design was not stated.

Meta-analyses, reviews, or other non-experimental studies.

### 2.5 Study screening and data extraction

We imported the bibliographies of the searched studies into EndNote20 software to check for duplicates and eliminate them. We then reviewed the titles and abstracts to exclude documents that did not meet the research objectives. Two authors independently selected studies based on the inclusion criteria, extracted the data using a standardized method, reviewed each other’s selected studies, and resolved disagreements through discussion or by consulting a third author.

The population in the literature was extracted following the PICOS principle—patient/population, intervention, comparison, outcome, and study design. The Hamilton Depression Scale (HAMD) was used to measure patient mental health, while the Fugl-Meyer Assessment (FMA) and Berg Balance Scale (BBS) were used to assess physical health. If the original study data were incomplete or poorly described, we attempted to contact the authors via email for supplementary information.

### 2.6 Quality assessment

The methodological quality and risk of bias in the included RCTs were assessed using the “Risk of bias” tool in Review Manager (RevMan) 5.4, provided by the Cochrane Collaboration Network. This assessment covered (1) random sequence generation; (2) allocation concealment; (3) blinding of participants and interventionists; (4) blinding of outcome evaluators; (5) completeness of outcome data; (6) selective reporting of research results; and (7) other potential sources of bias affecting the validity of the trials. Each evaluation was classified as “low risk,” “unclear,” and “high risk.” The risk of bias assessment was conducted independently by two review authors and cross-checked; disagreements were resolved through discussion or with the assistance of a third investigator. Literature with a grade C finding was excluded directly. Grade A/B/C was determined based on the ratio of low, uncertain, and high risk. If all criteria were low risk, the grade was A; if there was no high risk but some unclear risk, the grade was B; and if a high risk was present, the grade was C (19, 20).

### 2.7 Data analysis

Review Manage 5.4 software was used for statistical analysis. The experimental data were continuous, and the outcome measures included HAMD, FMA, and BBS. Therefore, mean deviation (MD) was used as the effect size, with a 95% confidence interval (95% CI) calculated. The effect size was interpreted based on Cohen’s criteria (21). A homogeneity test was conducted to assess heterogeneity among the studies, with *p* < α (test level *α* = 0.05) indicating heterogeneity between studies; conversely, studies were considered homogeneous. The *I*^2^ statistic was used to quantify the degree of heterogeneity, categorized into low, moderate, and high heterogeneity (22).

There are two result orientations in the Cochrane Handbook. If *I*^2^ ≤ 40%, a fixed-effect model was selected for meta-analysis. If *I*^2^ > 40%, a random-effects model was selected, and subgroup analyses were performed to identify sources of heterogeneity.

## 3 Results

### 3.1 Study search results

A total of 7,017 relevant articles were identified through the database search, with 912 articles from CNKI, 5,266 articles from Web of Science, 395 articles from the Wanfang Database, 157 articles from the VIP Database, and 27 articles from EBSCO. After removing 744 duplicate studies using the literature management software EndNote20, 6,273 studies remained.

The preliminary screening of titles and abstracts excluded 2,273 studies that were unrelated to the research content of this article. The remaining 1,979 studies were then screened further, excluding 2,021 articles that were found to be without full text.

**Figure 1:**
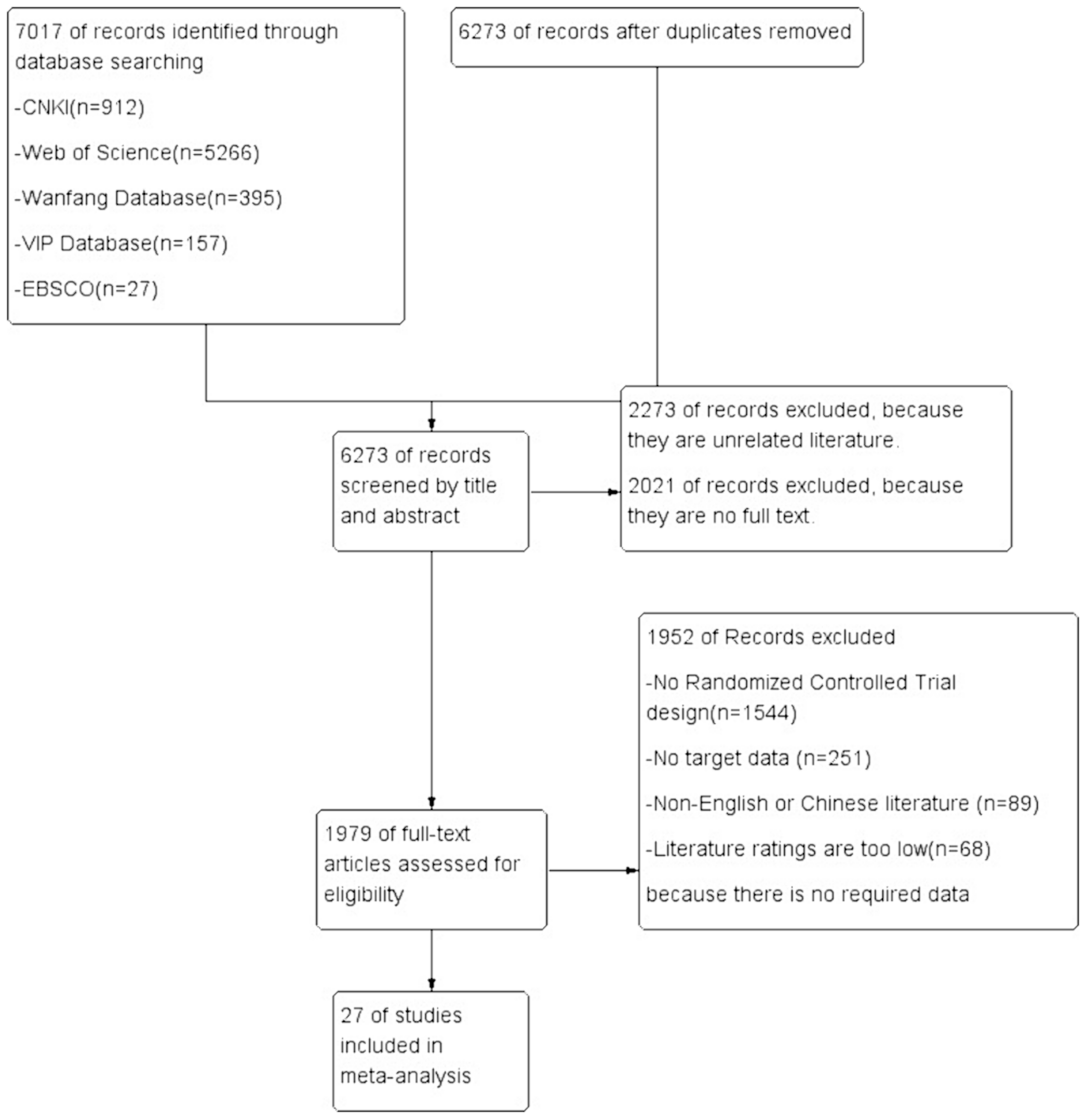
Literature screening flowchart.

Among these, 1,544 studies were excluded because they were not randomized controlled trials, 251 studies did not have the required data for literature, 89 studies were not in Chinese or English, and 68 studies were deemed of low quality. Finally, 27 studies were included to provide data support for this meta-analysis. This process highlights that, despite the large number of related studies, only a small proportion met the strict inclusion criteria. The literature selection process is shown in Figure 1.

### 3.2 Characteristics of the included studies

A total of 27 studies were included in this review, all of which were RCTs. Six studies reported outcomes using the HAMD, 13 studies used the FMA, and 16 studies used the BBS. The included studies provided sample sizes for both the experimental and control groups, with a total of 1,669 study participants: 853 participants in the experimental group and 816 participants in the control group. The mean age of participants was 58.37 years. The interventions used in the experimental groups included Tai Chi, Baduanjin, Yijinjing, Shierduanjin, and Wuqinxi Liuzijue, all based on conventional treatment.

Only two studies used “sitting” as the form of exercise, while the remaining studies involved standing exercises. A total of 13 studies did not specify whether exercises were conducted in groups or individually, seven studies explicitly stated group exercises, and the remaining six studies described individual exercises. All participants received professional supervision during the exercises according to the study protocols. The control groups received conventional treatment, which could include enhanced exercise relearning training or balance rehabilitation. The duration of a single intervention ranged from 12 to 60 min; with a frequency of 1 to 14 sessions per week, and the intervention periods varied from 3 to 20 weeks.

For detailed characteristics of the included studies, please see Table 1 (as both groups received conventional treatment, additional interventions beyond conventional treatment are detailed in the addendum).

**Table 1:** Included study characteristics table.

First author, year	Study design	Position	Exercise form	Patients *N* (male, female)		Age (years, M ± SD)		Intervention		Frequency of intervention	Outcome
		Stand/sit	Group /individual	Experimental group	Control group	Experimental group	Control group	Experimental group	Control group	Time/frequency/period	
Yuling Li, 2012 (23)	RCT	Sit	Individual	36 (17, 19)	32 (12, 20)	57 ± 6.32	57 ± 6.33	Tai Chi	No	30 min/ twice a week/8 weeks	HAMD
Xiaoyu Liu, 2021 (26)	RCT	Stand	Unclear	30 (11, 19)	30 (10, 20)	56 ± 7.47	57.58 ± 5.71	Baduanjin	No	48 min/3 times a week/4 weeks	HAMD/FMA/BBS
Shan Zhao, 2017 (27)	RCT	Stand	Group	30 (20, 10)	30 (19, 11)	53.85 ± 11.69	51.38 ± 14.83	Tai Chi	No	30 min/5 times a week/8 weeks	HAMD/FMA/BBS
Weicheng Zheng, 2015 (28)	RCT	Stand	Unclear	51 (27, 24)	55 (31, 24)	59 ± 13	60 ± 12	Tai Chi	No	30 min/7 times a week/12 weeks	HAMD
Xiaoyun Li, 2018 (29)	RCT	Stand	Group	30 (17, 13)	30 (22, 8)	71.03 ± 8.21	71.06 ± 8.33	Tai Chi	No	60 min/1 times a week/12 weeks	HAMD/FMA/BBS
Jing Du, 2021 (30)	RCT	Sit	Individual	30 (18, 12)	30 (17, 13)	50.57 ± 9.58	48.3 ± 8.18	Liuzijue	No	20 min/14times a week/4 weeks	HAMD
Pingping Sun, 2017 (33)	RCT	Stand	Unclear	30 (16, 14)	30 (20, 10)	63.73 ± 6.37	64.37 ± 5.74	Yijinjing	No	40 min/3 times a week/3 weeks	FMA
Linxia Tang, 2018 (34)	RCT	Stand	Unclear	30 (15, 15)	30 (14, 16)	59.2 ± 8.5	59.5 ± 8.8	Wuqinxi	No	60 min/5 times a week/20 weeks	FMA
Qiang Tang, 2022 (32)	RCT	Stand	Unclear	33 (21, 12)	34 (20, 14)	54.9 ± 13.1	56.5 ± 11.2	Tai Chi	Exercise relearning training	30 min/5 times a week/8 weeks	BBS
Hua Tian, 2017 (47)	RCT	Stand	Individual	30 (17, 13)	30 (19, 11)	54.3 ± 4.3	53 ± 4.3	Baduanjin	No	35 min/2 times a week/14 weeks	BBS
Chen Wang, 2022 (24)	RCT	Stand	Unclear	32 (24, 8)	31 (24, 7)	65.25 ± 9.21	60.65 ± 12.21	Liuzijue	Exercise relearning training	20 min/5 times a week/4 weeks	FMA/BBS
Jianping Wang, 2020 (48)	RCT	Stand	Group	30 (16, 14)	30 (17, 13)	55.97 ± 6.21	55.1 ± 6.28	Baduanjin	No	12 min/5 times a week/4 weeks	FMA
Meimei Wang, 2014 (35)	RCT	Stand	Unclear	30 (18, 12)	30 (19, 11)	49.3 ± 3.9	48.8 ± 4.3	Tai Chi	No	60 min/5 times a week/4 weeks	FMA/BBS
Peijing Xie, 2019 (36)	RCT	Stand	Unclear	20 (13, 7)	20 (12, 8)	51.1 ± 12.92	53.95 ± 13	Baduanjin	No	50 min/5 times a week/3 weeks	FMA/BBS
Qingxiang Zheng, 2019 (49)	RCT	Stand	Group	39 (24, 15)	39 (24, 15)	57.62 ± 10.76	60.97 ± 12.02	Baduanjin	No	20 min/14 times a week/6 weeks	FMA
Lingling Zhang, 2021 (50)	RCT	Stand	Unclear	41 (23, 18)	41 (24, 17)	71.29 ± 4.51	70.45 ± 4.29	Baduanjin	No	20 min/5 times a week/8 weeks	FMA
Yongsheng Cui, 2018 (51)	RCT	Stand	Unclear	24 (15, 9)	19 (12, 7)	53.67 ± 12.98	55.33 ± 14.32	Baduanjin	No	45 min/3 times a week/8 weeks	FMA
Weizong Jia, 2006 (52)	RCT	Stand	Group	18 (11, 7)	16 (9, 7)	47.7 ± 15.5	51.5 ± 16.37	Yijinjing	No	45 min/5 times a week/11 weeks	FMA/BBS
Lulu Niu, 2020 (53)	RCT	Stand	Unclear	40 (22, 18)	40 (21, 19)	62.75 ± 5.33	63.5 ± 5.14	Shierduanjin	No	30 min/14 times a week/8 weeks	BBS
Xiangbin Wang, 2016 (37)	RCT	Stand	Unclear	14 (9, 5)	16 (14, 2)	60.71 ± 7.32	58.56 ± 8.52	Tai Chi	Balance rehabilitation	60 min/5 times a week/12 weeks	BBS
Changxi Fu, 2016 (41)	RCT	Stand	Individual	30 (19, 11)	30 (18, 12)	59.7 ± 7.6	60.3 ± 8.4	Tai Chi	No	40 min/6 times a week/8 weeks	BBS
Xiaodong Xu, 2014 (54)	RCT	Stand	Unclear	40 (22, 18)	40 (16, 24)	60.14 ± 10.25	48.23 ± 12.32	Tai Chi	No	20 min/14 times a week/12 weeks	BBS
Li Dai, 2012 (55)	RCT	Stand	Group	48 (30, 18)	20 (9, 11)	68.1 ± 5.27	69.3 ± 5.91	Tai Chi	No	30 min/5 times a week/12 weeks	BBS
Senlin Guo, 2021 (56)	RCT	Stand	Group	14 (6, 8)	13 (6, 7)	64.71 ± 4.97	62.62 ± 5.32	Tai Chi	No	60 min/1 times a week/12 weeks	BBS
Jing He, 2022 (25)	RCT	Stand	Individual	29 (23, 6)	26 (20, 6)	62.96 ± 8.98	62.5 ± 10.73	Tai Chi	No	20 min/6 times a week/4 weeks	BBS
Kailiang Luo, 2022 (57)	RCT	Stand	Individual	24 (19, 5)	24 (19, 5)	66.25 ± 6.24	68.08 ± 5.9	Yijinjing	No	30 min/5 times a week/4 weeks	BBS
Zhibo Yang, 2012 (58)	RCT	Stand	Unclear	50 (35, 15)	50 (31, 19)	54.3 ± 13.8	55.2 ± 14.6	Tai Chi	Balance rehabilitation	45 min/6 times a week/4 weeks	BBS

### 3.3 Risk bias assessment

Risk bias assessment evaluates the quality of the included literature across seven evaluation dimensions: random sequence generation, allocation concealment, blinding of participants and personnel, blinding of outcome assessment, incomplete outcome data, selective reporting, and other bias. Can be selected from three of the seven latitudes of each article (low risk of bias, unclear risk of bias, and high risk of bias). Each study is assessed as having a low risk of bias, an unclear risk of bias, or a high risk of bias in each of these dimensions. In the seven evaluation categories for each article, only low and unclear risks of bias were identified—no instances of high risk of bias were found.

Among the 27 articles included, the assessment of random sequence generation found 24 studies with a low risk of bias and 3 studies with an unclear risk of bias. For allocation concealment, 13 studies were rated as low risk, 14 studies as unclear risk. The blinding of participants and personnel showed 2 studies with a low risk of bias and 25 studies with an unclear risk. For the blinding of outcome assessment, 11 studies had a low risk of bias, and 16 studies had an unclear risk. Incomplete outcome data were evaluated, with 25 studies showing a low risk of bias and 2 studies with an unclear risk of bias.

Selective reporting was assessed in all 27 studies, all of which were found to have a low risk of bias. Other biases were identified in 24 studies with a low risk of bias and 3 studies with an unclear risk. The unclear risks were primarily due to short study durations of only four weeks, while the general intervention time was around 8 weeks (24, 25), or practices that occurred only once a week for 60 min, compared to the usual frequency of about three times per week (56).

**Figure 2:**
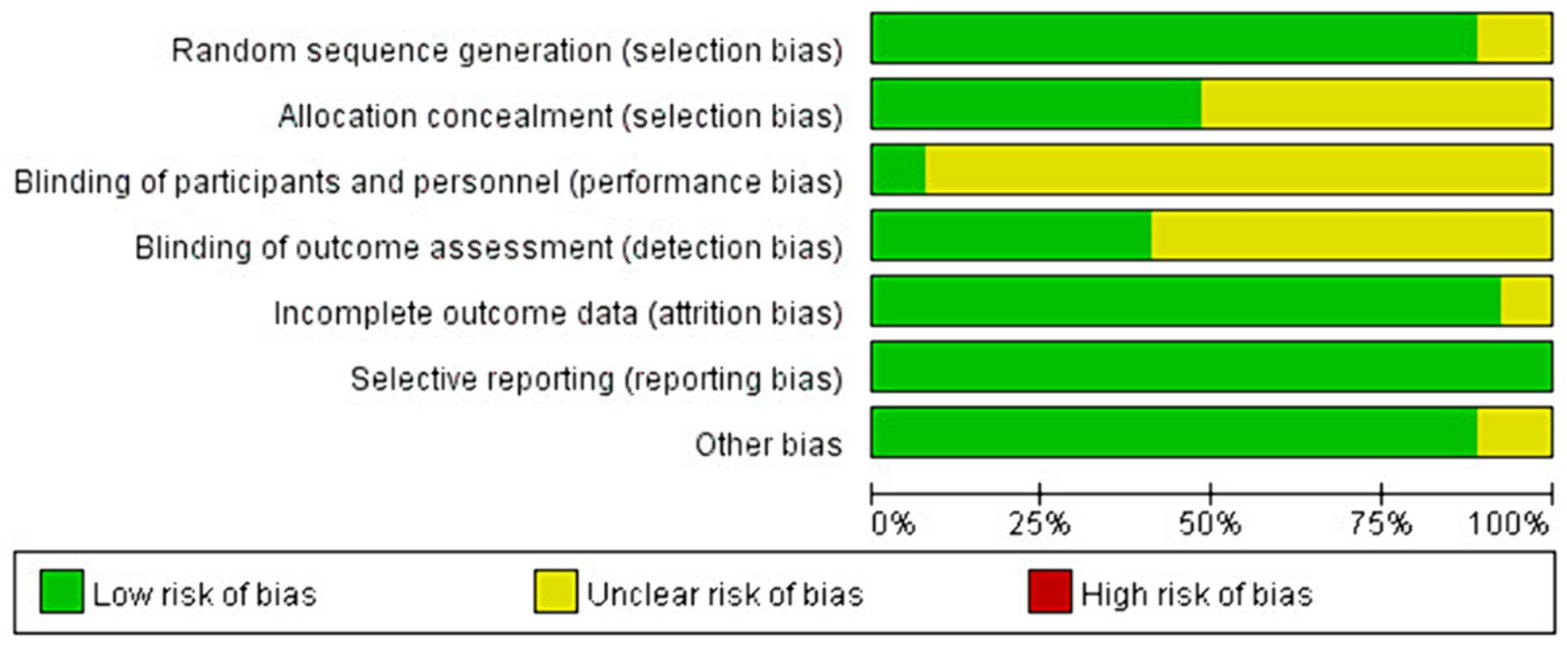
Risk bias assessment chart 1.

**Figure 3:**
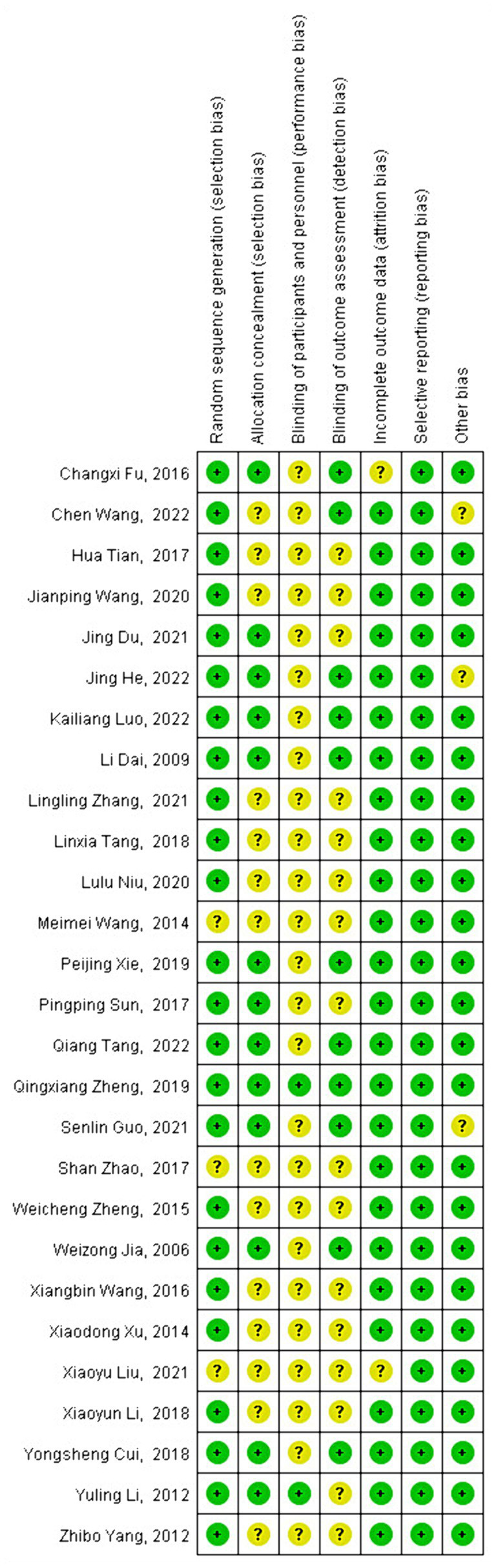
Risk bias assessment chart 2.

Out of the 27 studies, 24 studies used a random allocation method, and 12 studies described the allocation concealment scheme. Only 12 of the 14 included studies mentioned blinding. Data in 26 articles were complete, and no other significant biases were identified. Overall, the quality of the included studies was high. The most serious bias was related to the challenge of implementing double-blinding in exercise training interventions. Despite this, the overall risk of bias was low, indicating the high quality of the studies. The risk of bias in the included studies is detailed in Figures 2, 3.

### 3.4 Meta-analysis results and testing for heterogeneity

In the included studies, HAMD, FMA, and BBS were measured using the same method and units of representation, and MD was used as the pooled effect size for meta-analysis. Since only four studies in the control group performed other exercises, subgroup analysis was not conducted. The intervention duration (calculated as each session’s time multiplied by the number of weeks and the intervention cycle) was categorized into five groups: less than 500 min; 500–1,000 min; 1,000–1,500 min; 1,500–2,000 min, and more than 2,000 min.

Traditional Chinese sports were grouped according to different interventions, including Tai Chi, Baduanjin, Yijinjing, Shierduanjin, and Wuqinxi Liuzijue. Studies with high heterogeneity were subjected to subgroup analyses based on intervention timing and exercise programs to assess the dose-response relationship.

#### 3.4.1 Meta-analysis of HAMD effect size

A total of six studies analyzed the effect of traditional Chinese sports on HAMD scores. HAMD is an important reference indicator for measuring mental health, and the analysis showed that the HAMD scores of patients who practiced traditional Chinese sports significantly decreased [MD = −2.42, 95% CI: (−3.92 to −0.92), *p* = 0.002] compared to the control group. After testing for heterogeneity between the groups (*Q* = 26.8, df = 5, *I*^2^ = 81%, *p* < 0.001), a high level of heterogeneity was detected, so the analysis of the type and timing of traditional Chinese exercises was conducted using a random-effects model.

**Figure 4:**
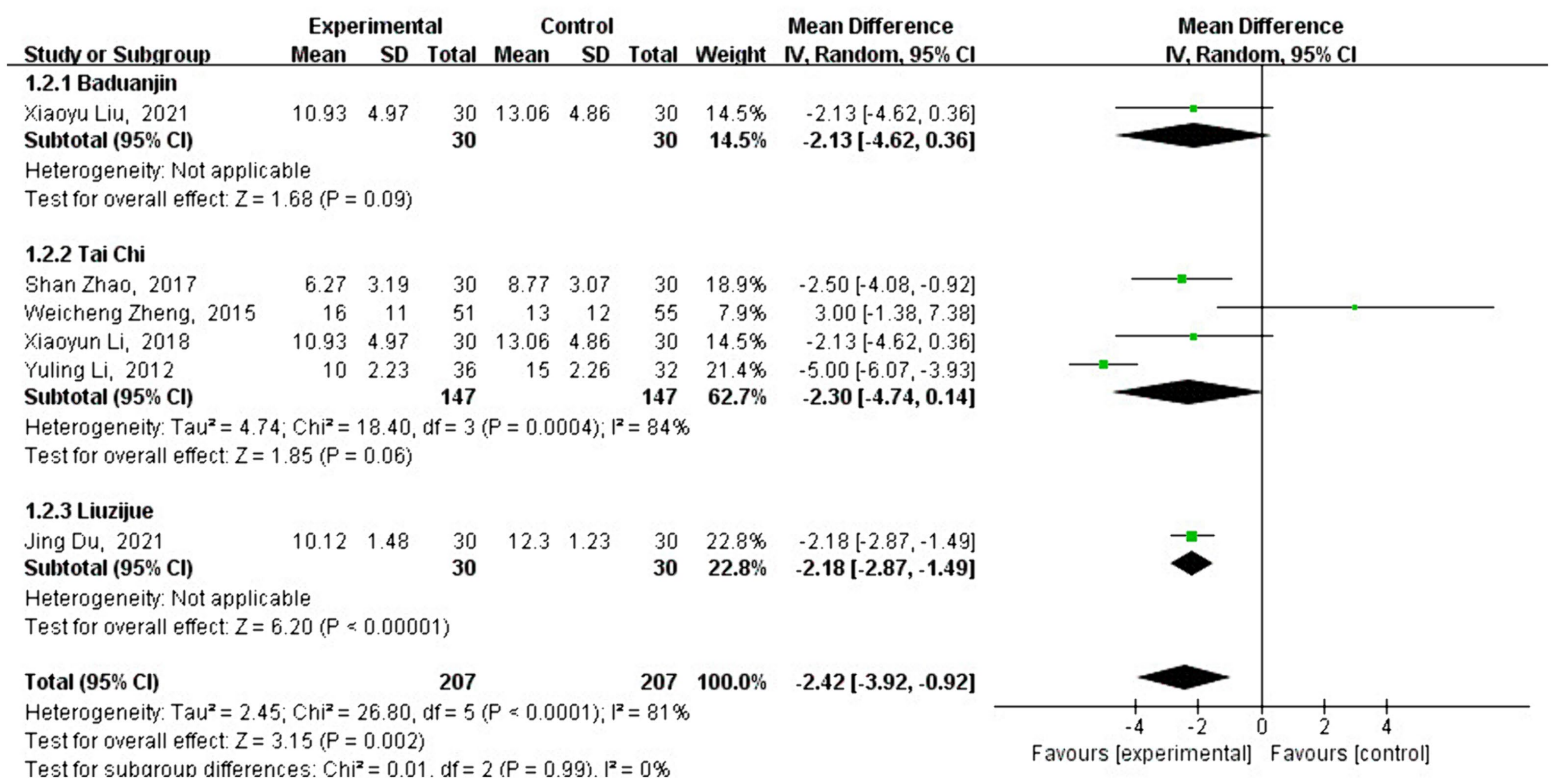
Intervention-time subgroup analysis of BBS effect sizes in forest plots.

**Figure 5:**
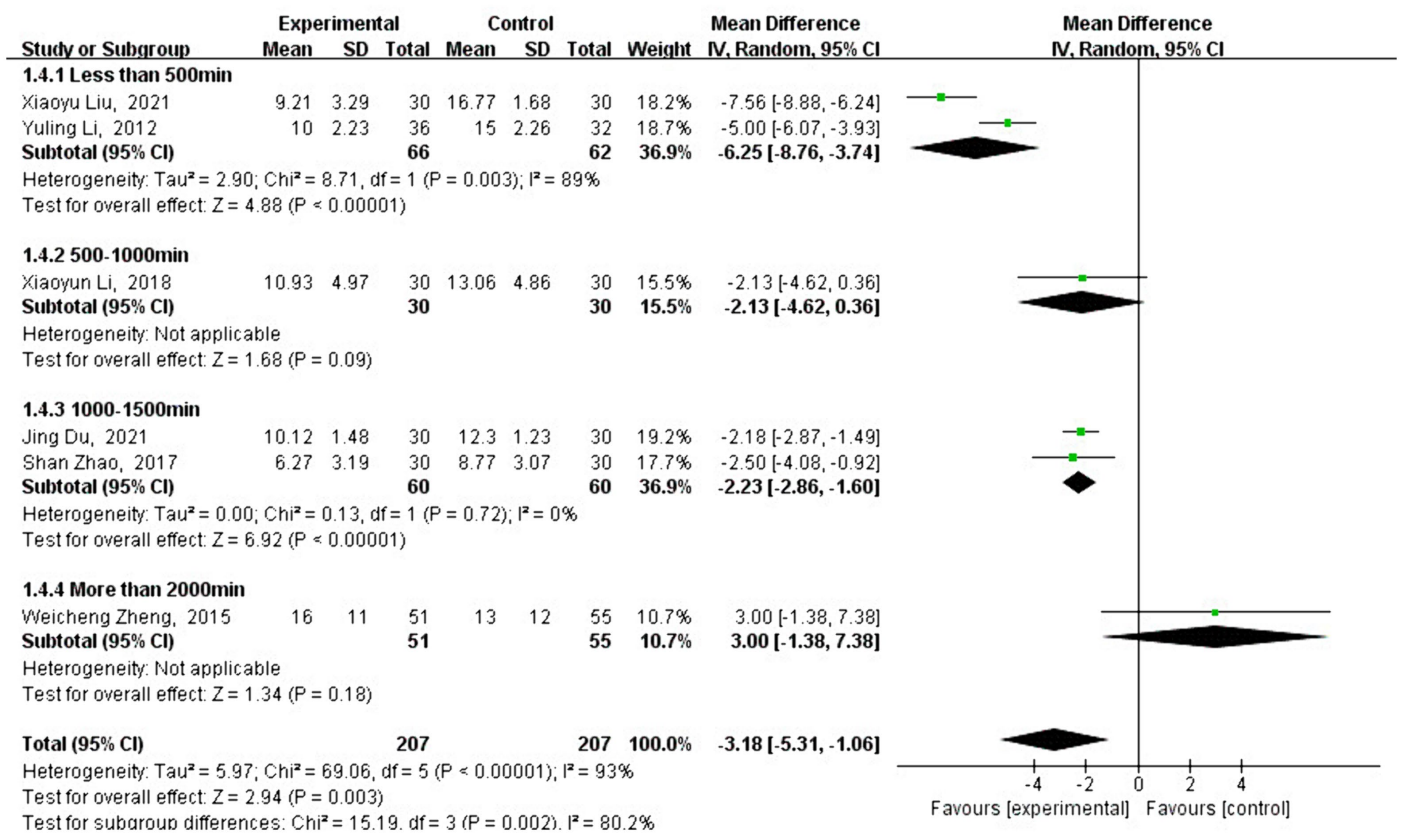
Intervention-type subgroup analysis of HAMD effect sizes in forest plots.

Subgroup analysis showed that the change in HAMD scores varied depending on the type of traditional Chinese exercise. The intervention using Liuzijue [MD = −2.18, 95% CI: (−2.87 to −1.49), *p* < 0.001] showed a significantly better effect than Baduanjin [MD = −2.13, 95% CI: (−4.62 to 0.36), *p* = 0.09] and Tai Chi [MD = −2.30, 95% CI: (−4.74 to 0.14), *p* = 0.06] (see Figure 4). As shown in Figure 5, different exercise durations also had a significant impact on HAMD scores. The most significant effects were observed in the groups with less than 500 min [MD = −6.25, 95% CI: (−8.76, −3.74), *p* < 0.001] and 1,000–1,500 min (MD = −2.23, 95% CI: [−2.86, −1.60), *p* < 0.001] of exercise. No studies were eligible for the 1,500 to 2,000 min group.

#### 3.4.2 Meta-analysis of FMA effect size

A total of 13 studies analyzed the effect of traditional Chinese sports on FMA intervention. FMA is a commonly used test to measure motor function, and the FMA scores of patients who engaged in traditional Chinese sports showed a significant increase [MD = 4.79, 95% CI: (4.14, 5.43), *p* < 0.001] compared with the control group.

After testing for heterogeneity between groups (*Q* = 84.34, df = 13, *I*^2^ = 85%, *p* < 0.001), a high level of heterogeneity was observed, promoting the use of a random-effects model to analyze the type and duration of traditional Chinese exercise. Subgroup analysis revealed that the change in FMA scores varied depending on the type of traditional Chinese exercise. Interventions using Baduanjin [MD = 4.35, 95% CI: (3.60, 5.10), *p* < 0.001], Yijinjing [MD = 6.57, 95% CI: (4.24, 8.89), *p* < 0.001], Wuqinxi [MD = 10.70, 95% CI: (6.17, 15.23), *p* < 0.001], and Tai Chi [MD =5.33, 95% CI: (3.76, 6.90), *p* < 0.001] were significantly more effective than no intervention.

**Figure 6:**
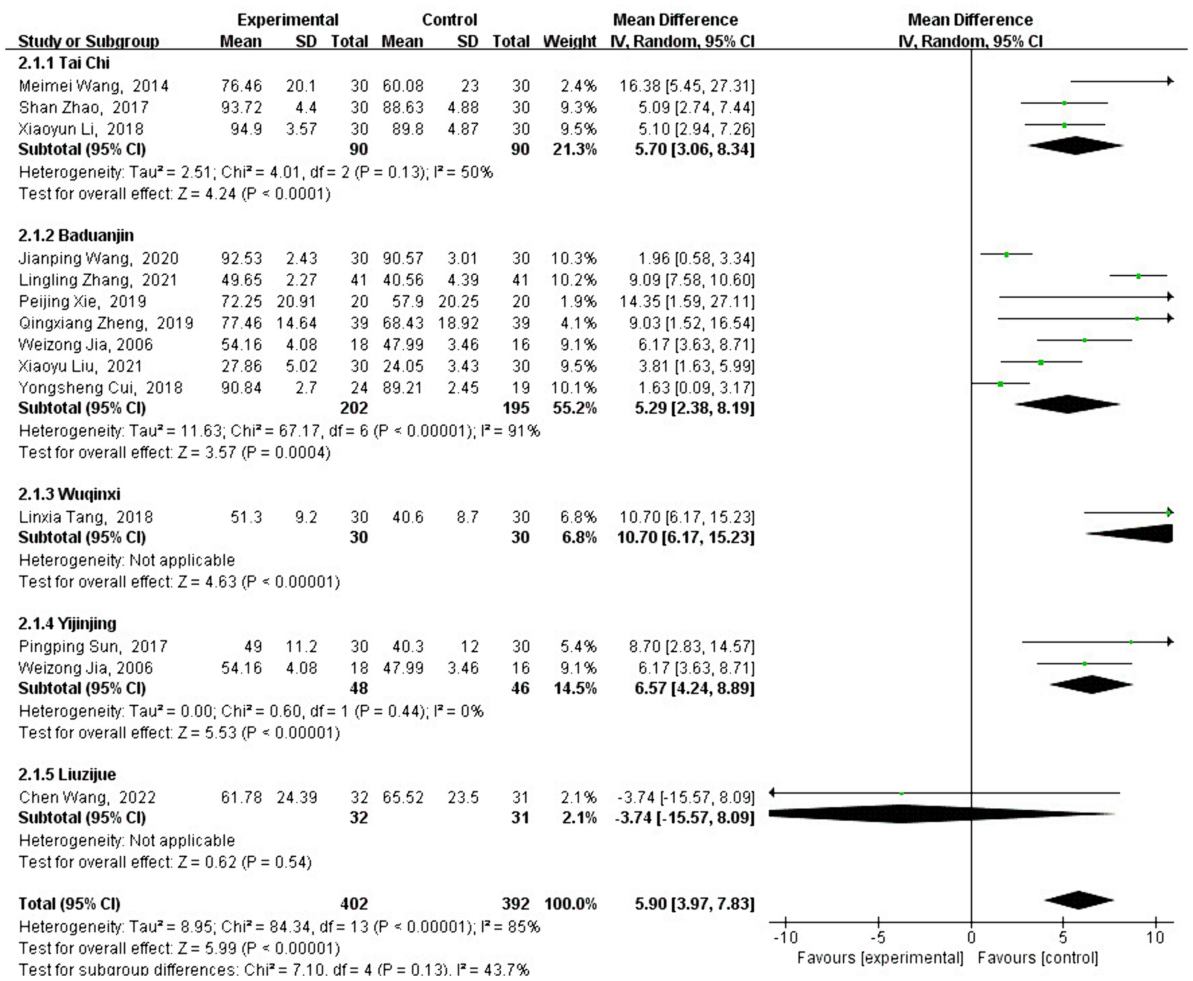
Intervention-type subgroup analysis of FMA effect sizes in forest plots.

**Figure 7:**
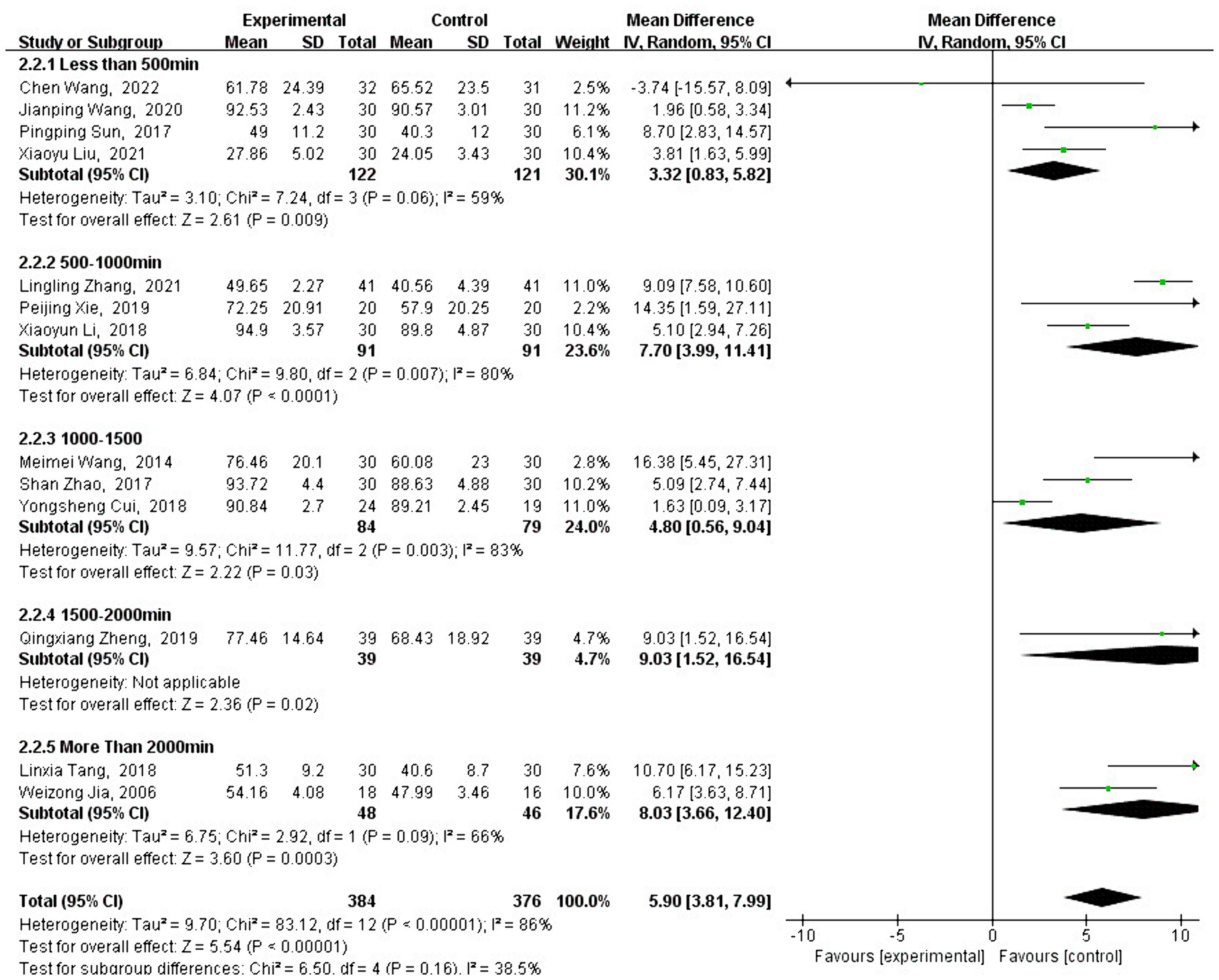
Intervention-time subgroup analysis of FMA effect sizes in forest plot.

However, Liuzijue [MD = −3.74, 95% CI: (−15.57, 8.09), *p* = 0.54] did not show a significant improvement compared to the control group (see Figure 6). As shown in Figure 7, different exercise durations also significantly impacted FMA scores, with 500–1,000 min [MD =7.70, 95% CI: (3.99, 11.41), *p* < 0.001] and more than 2,000 min [MD =8.03, 95% CI: (3.66, 12.40), *p* < 0.001] groups demonstrating the most significant improvements.

### 3.5 Meta-analysis of BBS effect size

A total of 16 studies analyzed the effect of traditional Chinese sports on BBS intervention. BBS is a widely used test to assess balance function, and the BBS scores of patients who participated in traditional Chinese sports significantly increased [MD = 5.25, 95% CI: (3.93, 6.56), *p* < 0.001] compared with the control group. After testing for heterogeneity between groups (*Q* = 164.47, df = 15, *I*^2^ = 91%, *p* < 0.001), high heterogeneity was identified, leading to the use of a random-effects model to analyze the type and duration of traditional Chinese exercise.

Subgroup analysis revealed that the change in FMA score varied depending on the type of traditional Chinese exercise selected. Interventions using Baduanjin [MD = 6.83, 95% CI: (1.55, 12.11), *p* = 0.01], Liuzijue [MD =4.58, 95% CI: (1.62, 7.54), *p* = 0.002], Shierduanjin [MD = 9.01, 95% CI: (6.71, 11.31), *p* < 0.001], and Tai Chi [MD = 4.99, 95% CI: (3.47, 6.51), *p* < 0.001] were significantly more effective than no intervention.

**Figure 8:**
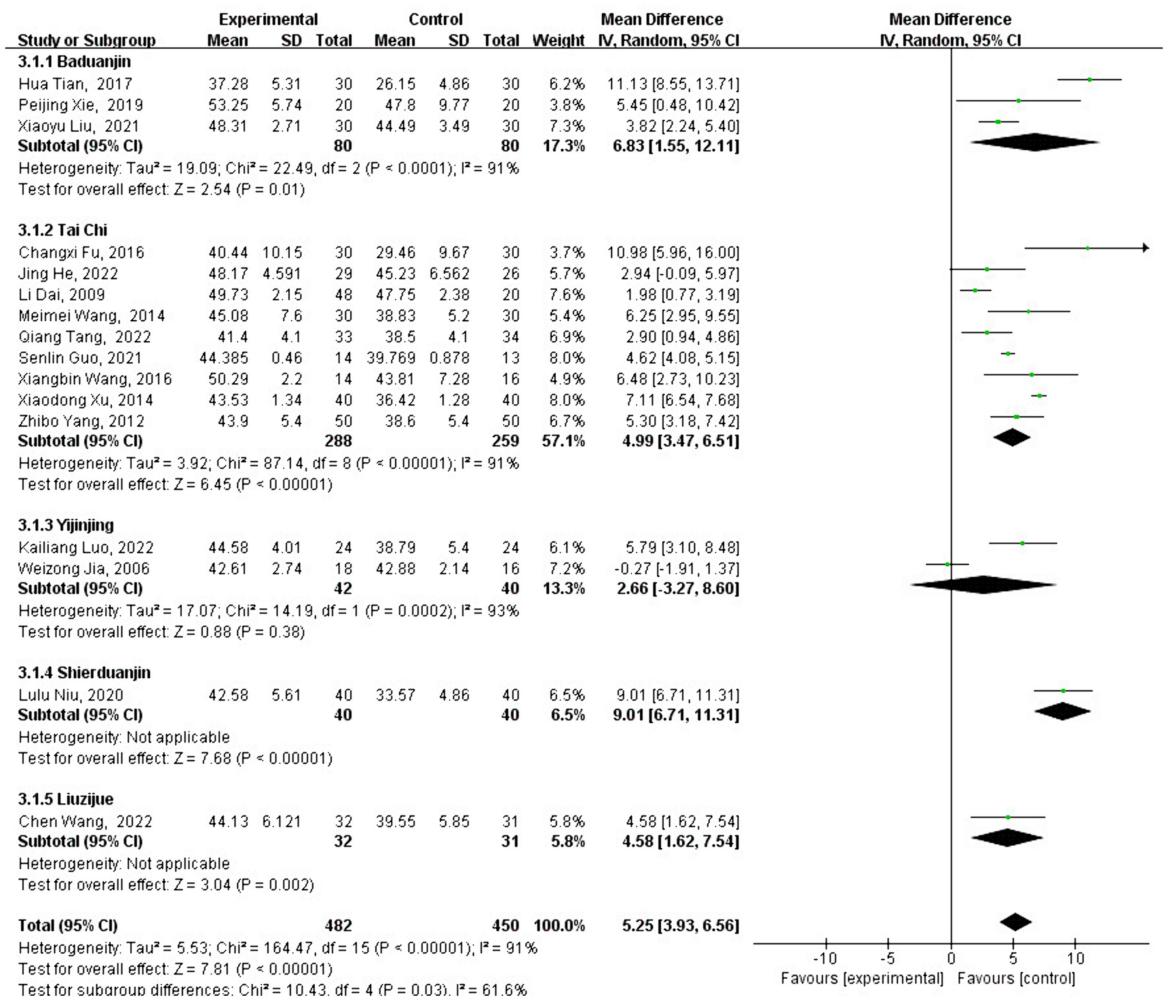
Intervention-type subgroup analysis of BBS effect sizes in forest plot.

**Figure 9:**
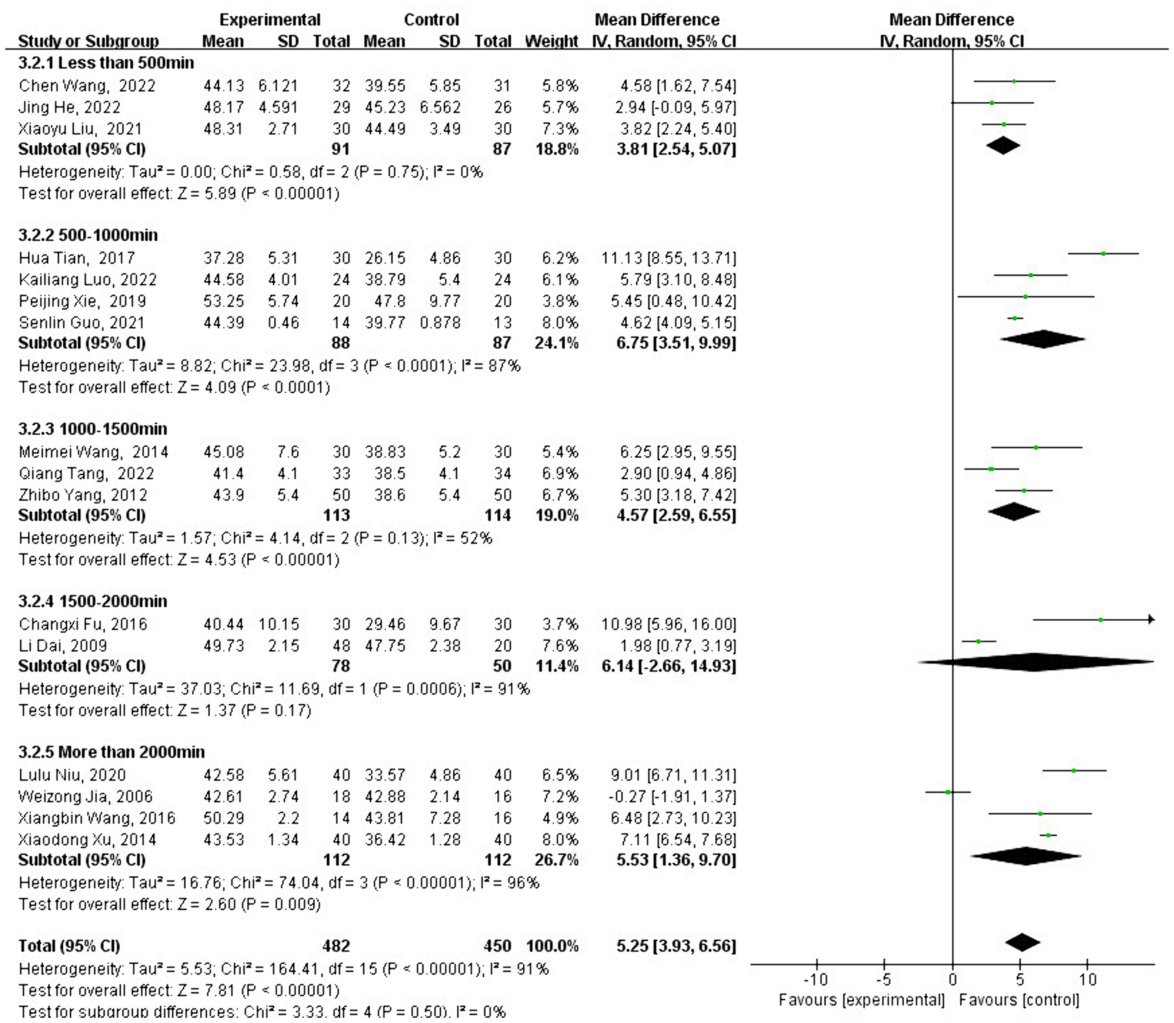
Intervention-time subgroup analysis of BBS effect sizes in forest plots.

However, Yijinjing [MD = 2.66, 95% CI: (−3.27, 8.60), *p* = 0.38] did not show a significant improvement compared to the control group (see Figure 8). As shown in Figure 9, different exercise durations also had a significant impact on BBS scores, with 500–1,000 min [MD =7.70, 95% CI: (3.99, 11.41) *p* < 0.001] and more than 2,000 min [MD =8.03, 95% CI: (3.66, 12.40), *p* < 0.001] groups showing the most significant impact.

### 3.6 Publication bias

**Figure 10:**
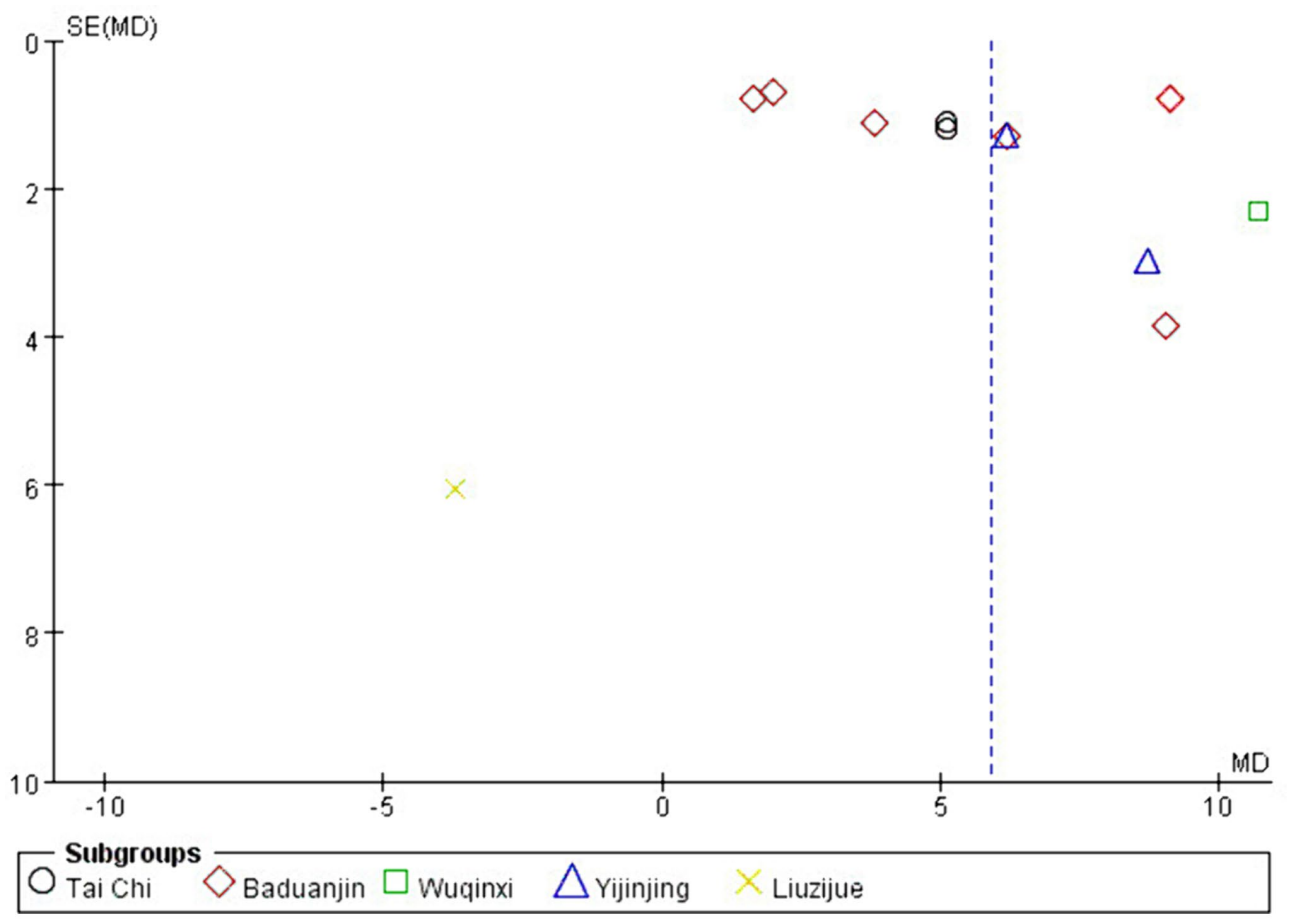
FMA funnel chart.

**Figure 11:**
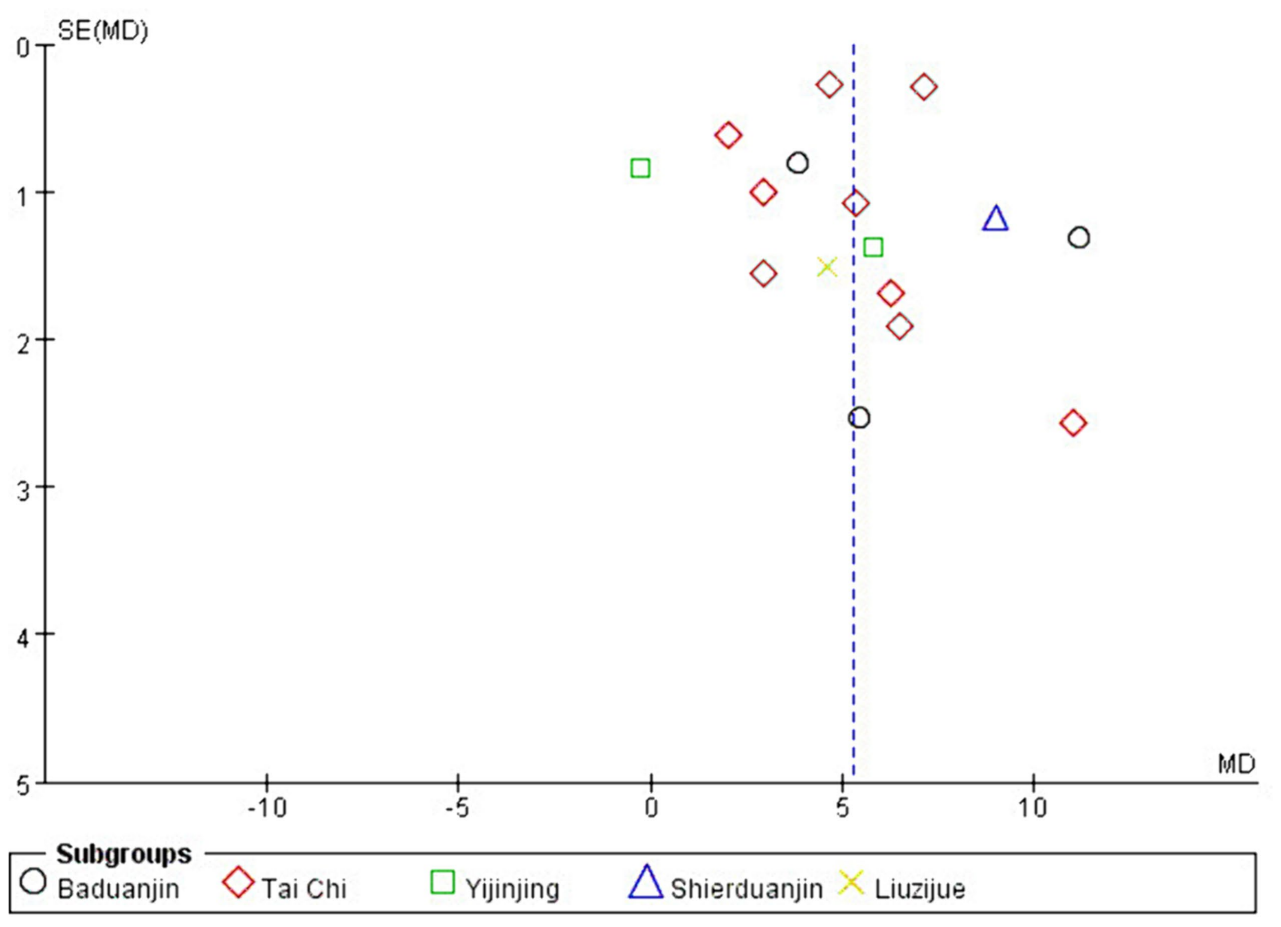
BBS funnel chart.

Publication bias refers to the possibility of biased data tendencies in certain types of study results, such as selectively reporting favorable outcomes to validate a study’s findings. In general, detecting publication bias is challenging when there are only a few studies of the same type. However, when the number of studies exceeds a certain threshold (typically more than 10), it becomes essential to conduct a publication bias test, as this helps assess the quality of the included data. In this study, there were fewer than 10 studies on HAMD effect sizes and more than 10 studies on FMA and BBS effect sizes, so funnel plots were prepared separately to evaluate publication bias. As shown in Figures 10, 11, the overall distribution of the included studies was relatively uniform, indicating no significant publication bias and suggesting that the study data are relatively reliable.

## 4 Discussion

This study primarily examines the impact of traditional Chinese exercises on the physical and mental health of stroke patients, specifically analyzing depression, exercise capacity, and balance using HAMD, FMA, and BBS as measurement indicators. The results of this study revealed that traditional Chinese exercises have a beneficial effect on the physical and mental health of stroke patients, which are discussed separately.

### 4.1 Traditional Chinese exercise can promote the mental health of stroke patients

The incidence of stroke is increasing each year, having a profound negative impact on patient psychology, recovery, and daily life. Therefore, exploring effective management strategies for post-stroke depression is crucial. According to contemporary medical understanding, post-stroke depression results from a combination of biological, psychological, and social factors, making its pathogenesis complex (23).

Studies have shown that an imbalance between free radical and anti-free radical defenses, along with decreased antioxidant capacity, may be the pathological basis leading to neurological and psychiatric disorders. Furthermore, exercise has been shown to enhance the antioxidant capacity of hippocampal tissue and upregulate the expression of brain-derived neurotrophic factors in the CA1 region of the hippocampus, offering protection against stroke-related depression (26).

Traditional Chinese exercises, classified as low-intensity aerobic exercises, are characterized by slow, soft, coordinated, and fluid movements combined with breathing exercises. These practices are believed to help unclog meridians, harmonize yin and yang, and regulate qi, leading to a state of natural relaxation where the body, mind, and breath are harmonized. This achieves the state of “quiet nothingness” and “spiritual inner guard.” Some studies suggest that the dynamic movements and stillness in Tai Chi and Qigong can induce a highly orderly state of brain relaxation, promoting the secretion of β-endorphins and contributing to mental relaxation. Additionally, the social interaction fostered by group Tai Chi exercises can further promote mental health (27–30).

The results of this study support the notion that practicing traditional Chinese exercises can indeed improve mental health, which is consistent with previous studies. Notably, the effect of practicing Liuzijue appears to be more pronounced, possibly due to its simple movements that allow practitioners to focus more on breathing and psychological conditioning. However, due to the limited number of relevant studies, this is only an inference.

### 4.2 Traditional Chinese exercise can promote physical health in stroke patients

Stroke patients often experience a decline in exercise ability and balance, and even during the recovery period, impaired mobility can be a significant obstacle to returning to normal life (31). As per some studies, exercise training is vital for stroke patients, particularly to address the abnormal manifestations of motor dysfunction and the loss of components during intensive rehabilitation (32). However, while rapid and vigorous exercise may more effectively activate superficial core muscles, their impact on deep core muscles is limited. Additionally, such exercises may aggravate spasms, leading to the solidification of movement patterns and a limited improvement in balance function. In contrast, the advantages of traditional Chinese exercise become more apparent in this context (24).

According to some studies, Yijinjing, which is rooted in the overall concept of traditional Chinese medicine and the theory of internal organs and meridians, can regulate emotions, improve the body’s defense mechanisms, and achieve the goals of invigorating righteousness, promoting a smooth qi and blood flow, and coordinating internal organs (33). Patients with stroke sequelae often choose Wuqinxi exercises to alleviate limb stiffness and pain, promote the recovery of motor function, improve daily activities, and improve quality of life. The mechanism behind these benefits may be related to brain plasticity and the improvement of brain structure and function (34).

Tai Chi mainly requires practitioners to relax their minds, focus on the coordination between movement and breathing, and move at a uniform speed, which helps reduce muscle tone and enables better voluntary control of the limbs. Additionally, the boxing routines in Tai Chi emphasize cooperation and alternating movements of both limbs, which can greatly improve limb coordination (35). However, Tai Chi exercises require a high level of physical coordination, making them difficult to learn, and it may be challenging for practitioners to gain confidence in the benefits of these exercises. In contrast, Baduanjin consists mostly of bilateral symmetrical movements, involves complex positioning, is simple and easy to learn, and can quickly build patients’ confidence in their recovery, making Baduanjin particularly suitable for clinical application in stroke patients (36).

In terms of balance ability, stroke patients typically exhibit an abnormal gait, often characterized by hemiplegic gait. Compared to healthy individuals, these patients show changes in temporal and spatial gait parameters, including reduced step length, and step frequency, along with increased step width. In addition, the duration of both the supporting phase and the swing phase of the healthy side is shortened, and the step length and stride length on the healthy side are significantly reduced compared to the affected side, leading to asymmetry in walking (affected side/healthy side) (37).

Studies have shown that Tai Chi, as a holistic exercise, emphasizes the combination of movement and stillness, requiring all movements to be performed with the body in a posture of a relaxed waist, dropped drop, and bent knees, allowing the center of gravity of the body to move in all directions, which greatly aids in improving balance function (38–40). Furthermore, Tai Chi training focuses on engaging patients, encouraging them to explore their potential. The exercise enhances human perception, particularly proprioception, by training muscle coordination, force, joint control, and nerve control, thereby restoring balance function to the greatest extent and effectively compensating for the limitations of modern rehabilitation technology (25, 41).

Yijinjing, another traditional exercise focused on improving motor function, involves slow and coherent movements. Most of these movements include static muscle exercises maintained through specific limb postures, which promote muscle strength and endurance. This practice can improve trunk balance and limb coordination (25).

The results of this study are consistent with previous studies, indicating that traditional Chinese exercises such as Tai Chi, Baduanjin, Wuqinxi, and Liuzijue can improve the physical health of stroke patients (42, 43). However, these exercises do not significantly improve the capacity of stroke patients but do have a notable effect on improving balance ability, which may be attributed to the relatively low exercise intensity of Liuzijue (44–46).

### 4.3 Limitations of existing research

We also found that, in terms of balance improvement, there is no clear dose-response relationship between exercise duration and effectiveness. In contrast, exercise capacity and mental health show an inverse relationship, where exercise durations exceeding 2,000 min significantly reduce the positive impact. This finding suggests that traditional Chinese exercises have bottlenecks and limitations in improving patients’ mental health and exercise capacity. Therefore, it is recommended that an intervention duration of approximately 2,000 min is most appropriate for enhancing mental health. For improving physical health, interventions should be conducted with patient safety as the priority, and currently, no clear dose-response relationship has been identified.

Although efforts were made to minimize data heterogeneity by applying explicit exclusion criteria, eliminating low-quality literature, and conducting subgroup analyses, some heterogeneity remains. This residual heterogeneity may be attributed to the varying baseline conditions of patients across the included studies. Although the overall results were positive, individual differences among patients contributed to this unavoidable heterogeneity.

To address this, low-quality studies were excluded through rigorous data quality evaluation. Additionally, for more extensively classified studies where the number of studies remained above three after subgroup classification, sensitivity analyses were conducted using the “one-by-one elimination method.” These analyses revealed low sensitivity, suggesting that the observed heterogeneity might stem from the immaturity of the research paradigm and significant variations in the original characteristics of the subjects.

In addition, most of the traditional Chinese exercises examined in existing studies are standing exercises. Given that many patients have limited mobility, future research should consider incorporating sitting exercises to accommodate those who are unable to stand. Furthermore, the experimental design of these exercise interventions requires greater rigor. Many current studies utilize single-blind or non-blinded groupings, and some studies do not specify whether the exercises were conducted one-on-one or in group settings. These aspects need to be addressed and strengthened in future studies.

This study also has its limitations, such as the lack of group discussion and comparison between group or individual exercises. Moreover, most of the included studies are from domestic sources, highlighting the need for further internationalization of research on traditional Chinese exercises.

## 5 Conclusion

Traditional Chinese exercise has a beneficial effect on the physical and mental health of stroke patients, with the most significant impact observed in balance function. However, the improvement in exercise ability and mental health does not necessarily increase with longer practice durations, as there appear to be certain limitations regarding the optimal duration.

Future research should explore the differences between various exercise methods, such as standing and sitting exercises, and provide detailed descriptions of the research methodologies to improve study quality, especially in terms of intervention methods. At present, most research on traditional Chinese sports is conducted by domestic experts; therefore, efforts should be made to promote these practices internationally and to engage the interest of foreign experts, which is a research direction worth pursuing.

## Data Availability

The raw data supporting the conclusions of this article will be made available by the authors, without undue reservation.
